# Complications of Endoscopic Ultrasound-Guided Fine Needle Aspiration: A Narrative Review

**DOI:** 10.3390/diagnostics10110964

**Published:** 2020-11-17

**Authors:** Masafumi Mizuide, Shomei Ryozawa, Akashi Fujita, Tomoya Ogawa, Hiromune Katsuda, Masahiro Suzuki, Tatsuya Noguchi, Yuki Tanisaka

**Affiliations:** Department of Gastroenterology, Saitama Medical University International Medical Center, 1397-1, Yamane, Hidaka, Saitama 350-1298, Japan; mizuide1971@yahoo.co.jp (M.M.); a.fujita0628@gmail.com (A.F.); t.ogawa0210@icloud.com (T.O.); hkatsuda0112@gmail.com (H.K.); msuzgast@tmd.ac.jp (M.S.); k055eb@yahoo.co.jp (T.N.); tanisaka1205@gmail.com (Y.T.)

**Keywords:** endoscopic ultrasound-guided fine-needle aspiration, complications, morbidity, mortality, pancreatitis, bleeding, infection, perforation, needle tract seeding

## Abstract

Considerable progress has been made recently in the use of endoscopic ultrasound-guided fine-needle aspiration (EUS-FNA) to diagnose intra-luminal gastrointestinal lesions and extra-luminal lesions near the gastrointestinal tract. Numerous reports have indicated that EUS-FNA has high diagnostic performance and safety, which has led to the routine use of EUS-FNA and an increasing number of cases. Thus, while EUS-FNA has a low rate of complications, endoscopists may encounter these complications as the number of cases increases. Infrequent reports have also described life-threatening complications. Therefore, endoscopists should possess a comprehensive understanding of the complications of EUS-FNA, which include hemorrhage, perforation, infection, and acute pancreatitis, as well as their management. This review examines the available evidence regarding the complications associated with EUS-FNA, and the findings will be useful for ensuring that endoscopists perform EUS-FNA safely and appropriately.

## 1. Introduction

Endoscopic ultrasound-guided fine-needle aspiration (EUS-FNA) was first reported in 1992 [1], and has subsequently developed into an important technique for diagnosing intraluminal gastrointestinal lesions, such as submucosal tumors (SMT) and gastrointestinal cancers [2]. Furthermore, EUS-FNA can be used for extra-luminal lesions that can be punctured from inside the gastrointestinal tract, such as lesions in the pancreas, spleen, adrenal glands, mediastinum, peritoneal cavity, intrapelvic organs, and nearby lymph nodes [3,4,5,6,7,8]. Moreover, EUS-FNA is useful for diagnosing malignant diseases before the selection of pharmaceutical or surgical treatment [9], as it can help determine whether the lesion is benign or malignant and its degree of progression [10,11]. Numerous reports have described the diagnostic performance and safety of EUS-FNA with various types of lesions [12,13,14], which has led to its broad acceptance among endoscopists. Thus, EUS-FNA is being performed in very high numbers at medical institutions. Although the complication rate of EUS-FNA is low, endoscopists may be increasingly be confronted with these complications based on the increasing number of patients. Therefore, this review covers the complications associated with EUS-FNA, with classification according to complication types and puncture targets.

## 2. Overview of Complications Associated with EUS-FNA

The major complications associated with EUS-FNA include hemorrhage, perforation, infection, and organ-specific complications, such as acute pancreatitis after puncture for pancreatic lesions. Recent reports have also occasionally described needle tract seeding (NTS) as a complication of EUS-FNA. While the specific rates vary according to lesion and study, the complication rate is approximately 0–2.5% [15,16,17,18,19,20,21,22,23,24,25,26,27,28,29,30,31], and the mortality rate is approximately 0.1–0.8% (Table 1). Deaths after EUS-FNA are generally related to fatal hemorrhage [15,32] or progression leading to death after duodenal perforation [27].

A previous systematic review of complications and deaths associated with EUS-FNA (51 reports and 10,941 patients) revealed a complication rate of 0.98% and a mortality rate of 0.02% [33]. The specific complication rates were 0.44% for acute pancreatitis, 0.34% for pain, 0.13% for hemorrhage, 0.11% for fever, 0.05% for infection, and 0.02% for perforation. The two deaths occurred in 1 case of severe acute pancreatitis (among 8246 patients with pancreatic lesions), and 1 case of severe cholangitis due to EUS-FNA performed for hepatic lesions (among 344 patients with hepatic lesions). Analyses according to puncture target revealed that the highest complication rate was observed when EUS-FNA was performed for ascites (3.53%), with lower complication rates when it was performed for hepatic lesions (2.33%) and in the rectal area (2.07%).

The safety of EUS-FNA for solid lesions has been reported numerous times, based on complication rates of 0–2.5% [19,20,21,22,24,28,29,31]. However, care is needed when performing EUS-FNA for cystic lesions, which have a higher rate of complications (13.6%) than pancreatic solid lesions and lymph nodes (0.5%) [16]. Another study examined the complications when EUS-FNA was performed for pancreatic solid lesions (134 patients) and pancreatic cystic lesions (114 patients) and revealed that complications only occurred in 4 patients with pancreatic cystic lesions (3.5%), including 3 cases of acute pancreatitis [18]. Moreover, another report indicated that 3 of 50 patients (6%) experienced hemorrhage after EUS-FNA was performed for pancreatic cystic lesions [34]. Nevertheless, these were small retrospective studies, and a prospective randomized study is needed to confirm whether cystic lesions are associated with a higher complication rate than solid lesions.

The EUS-FNA procedure can be performed using 19-gauge, 22-gauge, or 25-gauge needles, and some studies have compared the complication rates according to needle size. No obvious differences in complication rates were observed when 22-gauge needles (64 patients) and 25-gauge needles (67 patients) were used for pancreatic solid lesions [35], or when 22-gauge needles (57 patients) and 19-gauge needles (60 patients) were used for pancreatic solid lesions [36]. Recently, we can use Franseen and SharkCore needles for better tissue acquisition and diagnostic performance. The safety of using an FNB-needle is reported to be similar to that of using an FNA-needle [30,31]. Other studies have also indicated that there was no relationship between the number of needle passes and complications [18].

## 3. Types of Complications

### 3.1. Hemorrhage

Hemorrhage occurs in ≤2% of patients who undergo EUS-FNA [17,21,28,32], which generally involves minor hemorrhage at a gastrointestinal puncture that resolves spontaneously, and endoscopic hemostasis and blood transfusion are rarely required (0–0.44% of cases) [16,20,28,32]. There was no relationship between needle size and hemorrhage. A study of EUS-FNA using 19-gauge needle in 100 patients revealed no hemorrhage [29] and in a study to compare the complications of EUS-FNA using a 22-gauge needle with that using 19-gauge needle, there were no hemorrhages in either groups [36]. However, one study identified a EUS-FNA-associated hemorrhage outside the gastrointestinal wall in 3 of 227 patients (1.3%) [37], which was managed by inflating a balloon on the echoendoscope tip and compressing the puncture region for 15–20 min. None of those patients experienced the spread of the hemorrhage or any other major problems. Other proposed strategies for reducing the risk of hemorrhage spreading outside the gastrointestinal wall include a puncture that avoids the center of lesions that are supplied by large blood vessels, puncturing without passing through the lesion, and reducing the number of needle passes. Thus, although uncommon, it is important to be aware of EUS-FNA-associated hemorrhage potential, and the procedure should be discontinued immediately in the case of hemorrhages [34].

A few reports have described hemorrhage-related death. In one report, 1 patient (mortality rate: 0.48%) died 3 days after experiencing a hemorrhage from an inferior pancreaticoduodenal artery aneurysm, which developed 12 h after EUS-FNA was performed for a pancreatic lesion [15]. Another report described a patient with disseminated pancreatic cancer who experienced massive gastrointestinal hemorrhage at 6 h after EUS-FNA (mortality rate: 0.15%) [32]. An autopsy was performed for that patient, but the hemorrhage site was not identified.

Other reports have linked EUS-FNA-associated hemorrhages to antiplatelet agents and anticoagulatory agents. One study evaluated the risk of hemorrhage to be associated with EUS-FNA in 26 patients who received nonsteroidal anti-inflammatory drugs (NSAIDs, hemorrhage rate: 0%), 6 patients who received low-dose prophylactic heparin (hemorrhage rate: 33.3%), and 190 control patients who received no treatment (hemorrhage rate: 3.7%) [38]. Those results suggest that EUS-FNA can be performed safely for patients receiving oral aspirin or NSAIDs, although patients who receive low-dose prophylactic heparin may have an increased risk of hemorrhage, and discontinuation may be prudent. Similarly, the American Society for Gastrointestinal Endoscopy (ASGE) guidelines classify EUS-FNA as a high-risk procedure and recommend ceasing low-dose heparin administration at ≥8 h before EUS-FNA [39]. The risk of a EUS-FNA-associated hemorrhage also increases with the administration of oral anticoagulatory agents and/or thienopyridine antiplatelet agents, such as clopidogrel, and discontinuation of these drugs is recommended by various guidelines [40,41].

### 3.2. Infection

Three prospective studies of bacteremia associated with EUS-FNA in the upper gastrointestinal tract [42,43,44] revealed that 4 of 202 patients (2.0%) experienced transient bacteremia after EUS-FNA, although none of those patients experienced symptoms of infection. Thus, the risk of EUS-FNA-associated bacteremia appears to be similar to that for standard upper gastrointestinal endoscopy [44]. The lower gastrointestinal tract environment is different, which suggests that the risk of infection associated with EUS-FNA might also be different. However, a study of 100 patients who underwent EUS-FNA in the lower gastrointestinal tract only identified 1 patient (1.0%) with transient asymptomatic bacteremia after EUS-FNA [45].

The ASGE guidelines indicate that the risk of bacteremia due to EUS-FNA is low, and prophylactic antibiotics are not recommended when EUS-FNA is performed for solid lesions [46]. However, some reports have described mediastinitis after EUS-FNA that was performed for mediastinal cystic lesions [47,48,49,50], and infection after EUS-FNA that was performed for pancreatic cystic lesions [16,17]. Thus, there remains controversy regarding the risk of infection when EUS-FNA is performed for cystic lesions. Given the lack of large prospective studies, the ASGE guidelines merely suggest prophylactic antibiotic administration when EUS-FNA is performed for mediastinal and pancreatic cystic lesions. Very recently, A newly reported meta-analysis evaluated the efficacy of antibiotics prophylaxis prior to EUS-FNA of pancreatic cysts. The authors concluded that prophylactic antibiotics do not seem to substantially reduce the risk of infections after EUS-FNA of pancreatic cysts, and routine use of these should be questioned [51].

### 3.3. Gastrointestinal Perforation

Gastrointestinal perforation caused by the needle is uncommon, and a study into interventional endoscopic ultrasound (EUS), which included 224 punctures in 221 patients could only identify perforation in 1 patient (0.4%, third portion of the duodenum) [52]. Gastrointestinal perforation caused by the needle can often be treated conservatively using fasting, an intravenous drip, and antibiotic administration. Perforation is more commonly related to endoscopic manipulation, especially during diagnostic EUS, which typically causes a larger perforation that may require surgical management. This is related to the partially blind insertion of an echoendoscope with a long and rigid tip, but can also be related to an endoscopist’s limited experience, the patient’s anatomical characteristics, and/or the presence of gastrointestinal strictures and diverticula [53,54].

Cervical esophageal perforation reportedly occurs in 0.03–0.06% of cases that involve diagnostic EUS in the upper gastrointestinal tract [53,54]. A prospective study of 4,894 patients revealed that cervical esophageal perforation was found using a curvilinear echoendoscope in 3 patients (0.06%), who were all women in their 80s and had short webbed necks [54]. Thus, endoscopists should consider asking patients if they have a history of difficult endoscope insertion and using a wire-guided technique, with early diagnosis and prompt appropriate treatment of perforation if necessary. Another study revealed an esophageal perforation in 5 patients (0.15%) among 2518 patients who underwent diagnostic EUS, 670 patients who underwent EUS-FNA, and 136 patients who underwent EUS-guided intervention [32]. All 5 patients had esophageal strictures that were related to malignant tumors. Therefore, endoscopists should be aware of the potential for esophageal perforation due to manipulation in patients with esophageal strictures.

Duodenal perforation has been reported in 0.029–0.86% of cases [22,55,56,57], with mortality rates of 0.035–0.1% [27,57]. A study of 20,000 diagnostic EUS procedures identified 8 deaths, which were related to duodenal perforation caused by a curvilinear endoscope in 7 cases [57]. Four patients also had duodenal diverticula, which may be a risk factor for duodenal perforation [57].

There have been no large-scale studies regarding EUS-associated perforation of the lower gastrointestinal tract.

### 3.4. Acute Pancreatitis

Acute pancreatitis occurs in approximately 2% of cases that involve EUS-FNA [17,18,20,23,58]. A study of EUS-FNA in 4909 patients with pancreatic solid lesions revealed acute pancreatitis in 14 cases (0.29%), which involved mild pancreatitis in 10 patients, moderate pancreatitis in 3 patients, and severe pancreatitis in 1 patient [58]. The patient with severe pancreatitis died, which corresponds to a mortality rate of 0.02% [58]. In this context, acute pancreatitis develops because of puncture-related injury to the main pancreatic duct and its branches, which leads to pancreatic parenchymal swelling that occludes the pancreatic ducts [20]. Another study of EUS-FNA in 134 patients with pancreatic solid lesions and 114 patients with pancreatic cystic lesions revealed acute pancreatitis in 3 patients with cystic lesions in the pancreatic head or uncinate process [18]. Pancreatic cancer accounts for most pancreatic solid lesions, and sclerotic changes associated with the local inflammatory response may reduce the likelihood of developing acute pancreatitis. In addition, lesions in the pancreatic head or uncinate process require the endoscopist to navigate long distances through abundant pancreatic parenchyma to the puncture target site, which may increase the risk of developing acute pancreatitis [18]. Other risk factors for acute pancreatitis development include a history of acute pancreatitis within the previous 6 weeks [20] and puncture of benign lesions [58].

### 3.5. NTS

There have been several recent reports regarding NTS, which is an uncommon complication of EUS-FNA. Peritoneal dissemination after EUS-FNA for intraductal papillary mucinous carcinoma was first reported in 2003 [59]. Since that time, 28 cases of NTS have been reported (Table 2) [59,60,61,62,63,64,65,66,67,68,69,70,71,72,73,74,75,76,77,78,79,80,81,82,83].

The EUS-FNA puncture targets in the patients who developed NTS were pancreatic cancer in 21 patients, intraperitoneal lymph node metastasis from pancreatic head cancer in 1 patient, intraperitoneal lymph node metastasis from melanoma in 1 patient, mediastinal lymph node metastasis from gastric cancer in 1 patient, intraperitoneal recurrence of unknown primary cancer in 1 patient, solid pseudopapillary neoplasm in 1 patient, and intraductal papillary mucinous carcinoma in 2 patients. The transesophageal route was only used for 1 patient for a puncture, and the transgastric routine was used for the remaining 27 patients (Table 3).

All 21 patients with pancreatic cancer had tumors in the pancreatic body and tail, and the mean interval to the NTS diagnosis after curative resection was 24.8 months (range: 6–42 months) in 16 patients. Two patients had solid tumors that were identified in the posterior wall of the gastric body when surgery was performed after EUS-FNA, and NTS was diagnosed based on the resected specimen. However, the time from EUS-FNA to surgery was markedly different when preoperative chemotherapy was used (113 days), and when it was not used (25 days) [78]. In the case where NTS was diagnosed at 22 months after surgery, hemorrhage and adhesions were intraoperatively identified at the EUS-FNA puncture site, and a small number of tumor cells were observed within the lymphatic vessels in this region. It is possible that puncture during EUS-FNA can cause hemorrhage and adhesion development, which may influence the survival of tumor cells in the lymphatic vessels, and similar changes may occur within the gastric wall (Table 4) [65].

Fifteen patients had a recurrence that presented as gastric SMT-like masses, and the NTS diagnosis was performed via biopsy during upper gastrointestinal endoscopy or EUS-FNA. The principal modalities for guiding this diagnosis are computed tomography, 18F-fluorodeoxyglucose positron emission tomography (FDG-PET), and upper gastrointestinal endoscopy. However, the NTS was not identified using computed tomography or FDG-PET in 4 cases and was instead diagnosed based on upper gastrointestinal endoscopy findings. Therefore, if the puncture route is not included in the resected specimen, regular observation using upper gastrointestinal endoscopy is recommended for these patients [76,79]. It has also been suggested that an increase in CA19-9 concentration may facilitate the early detection of NTS [81]. Survival intervals of 40.5–62.4 months have been reported for patients who underwent curative resection, which highlights the importance of early detection and curative resection of NTS [79]. Interestingly, no cases of NTS were reported in one study of 126 patients who underwent EUS-FNA before pancreatic cancer surgery [84], while 6 cases of NTS (3.4%) were reported among 176 patients who underwent EUS-FNA before distal pancreatectomy for pancreatic body and tail cancer [79]. Thus, when EUS-FNA is performed in cases of resectable pancreatic body and tail cancer, it is essential to understand the risk of NTS and to reduce it as much as possible. It is obvious that EUS-FNA should not be performed when it does not guide treatment selection. However, in cases of the potentially resectable pancreatic body and tail cancer, where the EUS-FNA puncture route would not be contained within the resected region, measures to reduce the risk of NTS could involve avoiding EUS-FNA and/or reducing the number of needle passes as much as possible [68]. Nevertheless, a study of patients who underwent curative resection of malignant pancreatic solid tumors and cystic tumors revealed no differences in recurrence in the gastric wall and peritoneum according to whether preoperative EUS-FNA was performed, and the multivariate analysis failed to detect relationships between preoperative EUS-FNA and recurrence or shorter survival [9,67]. Furthermore, no differences in peritoneal dissemination frequency were observed according to the use of preoperative EUS-FNA in cases of intraductal papillary mucinous carcinoma [85]. Moreover, no significant differences were observed in recurrence-free survival or overall survival according to the use of preoperative EUS-FNA in cases that involved distal pancreatectomy for pancreatic body and tail cancer [79]. Therefore, the decision to use EUS-FNA should be based on the patient’s characteristics and the relative risks and benefits of the procedure.

## 4. Complications According to Puncture Target

### 4.1. Pancreatic Lesions

The reported complication rates are 0.5–2.5% when EUS-FNA is performed for pancreatic solid lesions [19,20,21,22,24]. The highest reported rate (2.5%) [21,24] included various complications, such as abdominal pain, nausea, and vomiting that were assessed at an emergency department, as well as over-sedation that required administration of a reversal agent. However, if complications are restricted to more serious conditions, such as hemorrhage, perforation, and acute pancreatitis, the reported complication rates decrease to 0.6% [21] and 1.1% [24]. Thus, the rate of major complications is ≤2% when EUS-FNA is performed for pancreatic solid tumors. Multivariate analysis revealed that a tumor diameter of ≤2 cm and neuroendocrine tumors were risk factors for complications after EUS-FNA [86].

A study of EUS-FNA revealed no complications among patients with pancreatic cystic lesions (approximately 60% of puncture targets for 1034 patients with pancreatic lesions), while acute pancreatitis and duodenal perforation were observed in some patients with solid lesions [27]. Other studies that only considered pancreatic cystic lesions revealed complication rates that were low and comparable to those for pancreatic solid lesions. For example, one study identified EUS-FNA-associated complications in 2 of 341 patients (0.6%) with cystic lesions [23], while another study identified complications in 13 of 603 patients (2.2%) [87]. Moreover, a systematic review and meta-analysis of EUS-FNA in patients with pancreatic cystic lesions (40 reports and 5124 patients) [88] revealed a morbidity rate of 2.7% and a mortality rate of 0.2%. Therefore, EUS-FNA appears to be a safe technique for pancreatic cystic lesions.

### 4.2. Mediastinal Lesions

The complication rate is low when EUS-FNA is performed for mediastinal lymph nodes and tumors. A study of EUS-FNA performed in 104 patients with suspected mediastinal lymph node metastasis from lung cancer metastases only identified a complication in 1 patient (0.96%, intra-procedural stridor) [89]. Another report of EUS-FNA performed in 213 patients with mediastinal lymph node lesions at low-volume EUS centers also only revealed an esophageal perforation in 2 patients (0.9%), which was treated conservatively [90]. A systematic review and meta-analysis of EUS-FNA for mediastinal lymph node lesions in patients with non-small cell lung cancer (18 reports and 1201 patients) only identified minor complications, such as sore throat and fever, in 10 patients (0.8%) [91]. When mediastinitis developed as a rare complication [47,48,49,50], the puncture targets were cystic lesions and necrotic lymph nodes. Therefore, care is needed when considering these puncture targets.

### 4.3. Intrapulmonary Lesions

A systematic review and meta-analysis evaluated transesophageal EUS-FNA performed for intrapulmonary tumors (11 reports and 313 patients) [92]. That study included one report of 256 patients with available complication-related data, and only 5 patients (2.0%) experienced minor complications, such as pneumothorax and hemoptysis. None of the patients experienced severe complications, such as mediastinitis, major hemorrhage, or esophageal perforation.

### 4.4. Lymph Nodes

One study identified EUS-FNA-associated complications in only 1 of 130 patients with intraperitoneal lymph node swelling [93]. Similarly, a different report described mild abdominal pain in 3 of 147 patients (2.0%) with intraperitoneal lymph node swelling and no cases that involved severe complications, such as hemorrhage or infection [94]. Another study revealed only 1 case (1.0%) of mild abdominal pain that developed after EUS-FNA was performed in 104 patients with lymph node lesions, including 50 patients with mediastinal lymph node lesions and 48 patients with intraperitoneal lymph node lesions (1.0%) [25]. Therefore, EUS-FNA appears to be a safe technique for intraperitoneal and mediastinal lymph node lesions.

### 4.5. Intrapelvic Lesions

Among 29 patients with intrapelvic lesions, 2 patients (6.9%) developed abscesses requiring percutaneous drainage after EUS-FNA was performed via the lower gastrointestinal tract [95]. Both patients who developed abscesses had undergone EUS-FNA for cystic lesions. Thus, similar to cases of mediastinal cystic lesions, which carry a risk of mediastinitis, the possibility of infection should be considered when performing EUS-FNA for intrapelvic cystic lesions. It has been suggested that EUS-FNA should be avoided if the intrapelvic cystic lesions can be monitored via imaging and that EUS-FNA should be reserved for suspected malignant lesions and/or in cases where the treatment strategy is being changed. Nevertheless, no complications were observed among 20 patients, including 5 patients with cystic lesions who underwent EUS-FNA for intrapelvic lesions [8]. However, that study involved prophylactic antibiotics in cases that involved cystic lesions and solid lesions that required numerous punctures, which might have helped prevent infection.

### 4.6. Gastrointestinal SMT

Numerous studies have shown that when EUS-FNA is performed for upper gastrointestinal SMT, the technique is safe, and the complication rate is low [1]. However, no large studies have evaluated EUS-FNA for lower gastrointestinal SMT, although one small study failed to detect complications in 10 patients who underwent EUS-FNA for lower gastrointestinal SMT [96]. In another study of 502 patients who underwent EUS-FNA via the lower gastrointestinal tract, multivariate analysis revealed that puncture within the gastrointestinal wall was not a risk factor for complications [97]. Thus, EUS-FNA can likely be performed safely for SMT within the gastrointestinal wall, even if the lesion is located in the lower gastrointestinal tract. Another study of 1135 patients who underwent EUS-FNA for gastrointestinal SMT revealed that only 5 patients (0.44%) developed a hemorrhage requiring endoscopic hemostasis and blood transfusion, and no patients experienced perforation [28]. Some reports have described abscess formation leading to severe sepsis after EUS-Tru-Cut needle biopsy (using Tru-Cut needles) for gastric SMT [98] and abscesses requiring drainage after EUS-FNA for duodenal SMT [99]. Thus, it has been suggested that the Tru-Cut needles might cause major intra-tissue damage that might lead to intra-tissue hemorrhages and an environment that favors bacterial proliferation, with repeated punctures potentially being linked to the onset of severe infection.

Recent attempts have been made to perform EUS fine-needle biopsy (EUS-FNB) using the franseen-type needle (Acquire; Boston Scientific, Marlborough, MA, USA) and the fork-tip needle (SharkCore; Medtronic, Newton, Mass and Covidien, Dublin, Ireland). This procedure aims to provide better sample acquisition and diagnostic performance, and the safety is reportedly similar to that of EUS-FNA. A study comparing FNA and FNB revealed complications in only 1 of 229 cases (0.4%), which involved 1 case of hemorrhage (requiring endoscopic hemostasis) among 115 patients in the EUS-FNA group and no complications among the 114 patients in the EUS-FNB group [31]. Similarly, no complications were observed among 44 patients who underwent EUS-FNA and 17 patients who underwent EUS-FNB for gastrointestinal SMT [100]. A meta-analysis also compared EUS-FNA and EUS-FNB for gastrointestinal SMT (10 reports and 669 patients) [101], which revealed complications in 3 patients in the EUS-FNA group and 3 patients in the EUS-FNB group. However, those complications involved minor hemorrhage and aspiration pneumonia, which did not influence the patient’s treatment.

### 4.7. Hepatic Lesions

The reported complication rate is 0–3.6% when EUS-FNA is performed for hepatic lesions [102,103,104]. A single-center study revealed no complications among 77 patients who underwent EUS-FNA for hepatic lesions [102], and another study failed to detect complications among 47 patients who underwent EUS-FNA for hepatic tumors, including 17 patients with right lobe tumors [103]. However, another study identified complications in 6 of 167 patients (3.6%) who underwent EUS-FNA for hepatic lesions [104]. These patients included 2 patients who developed abdominal pain that spontaneously resolved, 2 patients who developed a fever but did not require antibiotic treatment, and 1 patient who experienced a hemorrhage which spontaneously resolved. However, 1 patient (0.6%) developed severe cholangitis and ultimately died because of sepsis. That patient had obstructive jaundice, and it was suggested that bacteria might have invaded the obstructed bile duct via the puncture. Thus, it is possible that biliary drainage should have been performed before EUS-FNA.

### 4.8. Biliary Strictures and Gallbladder Lesions

Two systematic reviews and meta-analyses have evaluated EUS-FNA for bile duct and gallbladder lesions. The first meta-analysis (9 reports and 284 patients) revealed no complications among patients with bile duct strictures and gallbladder tumors, which suggested that EUS-FNA is a safe and useful tool for diagnosing bile duct lesions and gallbladder tumors [105]. The second meta-analysis (20 reports and 957 patients with malignant bile duct strictures) revealed that 4 patients experienced complications among 383 patients in 11 studies. Three cases involved a minor hemorrhage that spontaneously resolved, and 1 case involved severe biliary peritonitis that resulted in death, which corresponded to an overall complication rate of 1.0% and a severe complication rate of 0.3% [106].

Endoscopic retrograde cholangiopancreatography (ERCP) and EUS-FNA have been compared as the main techniques for diagnosing biliary diseases. One study identified no complications among 16 patients who underwent EUS-FNA for gallbladder lesions, while mild acute pancreatitis was observed for 5 of 25 patients (20%) who underwent endoscopic transpapillary gallbladder drainage [107]. Another study of patients with extrahepatic bile duct lesions revealed no complications among the 19 patients who underwent EUS-FNA, although complications were observed for 14 of 54 patients (25.9%) who underwent ERCP, including 1 case of severe acute pancreatitis [108]. Thus, it appears that the complication rate is substantially higher when tissue is acquired using ERCP.

Peritoneal dissemination can occur when percutaneous puncture or EUS-FNA is performed for hilar cholangiocarcinoma. Thus, performing FNA via the intraperitoneal route should be avoided when radical resection is feasible [109]. There is also a risk of peritoneal dissemination via biliary leakage when EUS-FNA is performed for malignant bile duct and gallbladder lesions, although the use or non-use of preoperative EUS-FNA for bile duct cancer did not influence overall survival or progression-free survival [110]. Thus, it appears that the use of EUS-FNA does not influence the postoperative prognosis in this setting.

### 4.9. Adrenal Lesions

A single-center study of EUS-FNA in 121 patients with adrenal lesions revealed no complications that were associated with EUS-FNA, although percutaneous puncture had a complication rate of 4% at the same center [111]. Another study identified no complications among 59 patients who underwent EUS-FNA for adrenal lesions, including 5 patients with right adrenal lesions [112]. In this setting, EUS-FNA appears to be safe for an adrenal puncture, as only the gastrointestinal wall is interposed, while percutaneous puncture risks injury to the pleura, spleen, pancreas, and other organs [112]. Thus, EUS-FNA appears to be a safe option for adrenal lesions.

A multicenter study compared tissue acquisition using EUS-FNA or EUS-FNB (204 adrenal regions in 200 patients) and revealed that only 1 patient (0.5%) developed a fever, with a negative blood culture result [5]. None of the patients developed severe complications, and the use of FNB did not increase the complication rate [5]. These findings suggest that the use of a 22-gauge or 25-gauge needle instead of a 19-gauge needle might help reduce the complication rate. No hypertensive crises occurred in that study, although 2 patients had pheochromocytoma, which could not be definitively diagnosed based on the lack of pre-procedural testing. Thus, regardless of whether there is suspected adrenal metastasis, the differential diagnosis should consider pheochromocytoma to ensure patient safety. Moreover, although EUS-FNA is a fundamentally safe technique for adrenal lesions, adrenal hemorrhage has been reported [113], and computed tomography should be performed to investigate abdominal pain that develops after EUS-FNA.

### 4.10. Splenic Lesions

Few reports have addressed EUS-FNA for splenic lesions, and these reports generally involved single cases or small sample sizes. One study evaluated 12 patients who underwent EUS-FNA because of lesions that were difficult to puncture (e.g., small lesions) or after a diagnosis could not be achieved via computed tomography or EUS-guided puncture [114]. That study indicated that 1 patient (8.3%) developed abdominal pain, but none of the patients experienced hemorrhage or other severe complications [114]. Another report did not identify any hemorrhage or severe complications among 5 patients who underwent EUS-FNA with a 19-gauge needle for splenic lesions [4]. Furthermore, no complications were described in a report of 36 patients who underwent EUS-FNA for splenic lesions, including 12 tumorous lesions [115] or in another report of 15 patients who underwent EUS-FNA for splenic lesions [116]. However, among 16 patients who underwent EUS-FNA for splenic lesions, including 8 cystic lesions, 1 patient experienced massive gastrointestinal hemorrhage at 7 days after the procedure, although the hemorrhage did not appear to be causally related to the EUS-FNA [117]. That patient had a pseudocyst, and it is possible that the hemorrhage was related to a splenic artery pseudoaneurysm [117]. There do not appear to be any reports of infection after EUS-FNA for splenic lesions, even in cases that involved cystic lesions.

## 5. Conclusions

As a method for tissue collection, EUS-FNA plays an important role for the diagnosis of various lesions with a tolerable complication rate. Although EUS-FNA has a low rate of complications, severe complications are possible. Therefore endoscopists must know the latest information regarding the complications associated with EUS-FNA and be prepared to manage these complications.

## Figures and Tables

**Table 1:** An overview of the complications associated with endoscopic ultrasound-guided fine needle aspiration.

First Author	Year	Ref.	Number of Cases	Puncture Target	Complication Rate, % (n)	Mortality Rate, % (n)	Details
Gress, F.G.	1997	[15]	208	Mixed: mediastinal lymph nodes, intra-abdominal lymph nodes, pancreatic lesions, submucosal masses, and perirectal masses	1.9% (n = 4)	0.8% (n = 1)	All 4 patients had pancreatic lesions as the puncture target: 2 patients experienced acute pancreatitis, and 2 patients experienced hemorrhage (fatal in 1 case).
Wiersema, M.J.	1997	[16]	457	Mixed: solid lesions (lymph nodes, extraluminal masses, gastrointestinal wall lesions) and cystic lesions	1.1% (n = 5)	0.0% (n = 0)	Solid-lesion complications: 0.5% (2/435), cystic-lesion complications: 13.6% (3/22).
Williams, D.B.	1999	[17]	333	Mixed: lymph nodes, pancreatic lesions, extraintestinal masses, and intramural tumors	0.3% (n = 1)	0.0% (n = 0)	Streptococcal bacteremia was confirmed via EUS-FNA in pancreatic tail cystic lesions, which resolved after conservative treatment.
O’Toole, D.	2001	[18]	322	Mixed: pancreatic solid lesions, pancreatic cystic lesions, lymph node, stromal tumors, and others	1.6% (n = 5)	0.0% (n = 0)	Pancreatic solid lesion and lymph node complications: 0%, pancreatic cystic lesion complications: 3.5% (4/114; acute pancreatitis: 3 cases, aspiration pneumonia: 1 case)
Harewood, G.C	2002	[19]	185	Pancreatic solid masses	0.5% (n = 1)	0.0% (n = 0)	Mild acute pancreatitis: 1 case.
Gress, F.G.	2002	[20]	100	Pancreatic solid masses	2.0% (n = 2)	0.0% (n = 0)	Mild acute pancreatitis: 2 cases (both had histories of acute pancreatitis).
Eloubeidi, M.A.	2003	[21]	158	Pancreatic solid masses	2.5% (n = 4)	0.0% (n = 0)	Mild acute pancreatitis: 1 case (history of acute pancreatitis), abdominal pain examined at the emergency department: 1 case, nausea and vomiting: 1 case, over-sedation: 1 case.
Raut, C.P.	2003	[22]	233	Pancreatic solid masses	1.7% (n = 4)	0.0% (n = 0)	Duodenal perforation: 2 cases (required surgery), acute pancreatitis: 1 case, abdominal pain: 1 case.
Brugge, W.R.	2004	[23]	341	Pancreatic cystic lesions	0.6% (n = 2)	0.0% (n = 0)	Mild acute pancreatitis: 2 cases (single puncture for each patient using 22- and 19-gauge needles).
Eloubeidi, M.A.	2006	[24]	355	Pancreatic solid masses	2.5% (n = 9)	0.0% (n = 0)	Acute pancreatitis: 3 cases, infection: 1 case (surgical debridement for necrosis), fever: 1 case (no infection), abdominal pain: 3 cases, over-sedation: 1 case.
Yasuda, I.	2006	[25]	104	Mixed: mediastinal lymph nodes and intra-abdominal lymph nodes	1.0% (n = 1)	0.0% (n = 0)	Abdominal pain: 1 case.
Al-Haddad, M.	2008	[26]	483	Mixed: pancreatic solid lesions, pancreatic cystic lesions, lymph nodes, hepatic lesions, biliary lesions, submucosal masses, and pelvic masses	1.4% (n = 7)	0.0% (n = 0)	Abdominal pain: 4 cases, chest pain: 1 case, melena: 1 case, fever: 1 case (no infection).
Carrara, S.	2010	[27]	1034	Pancreatic solid or cystic lesions (~40% were solid masses)	0.3% (n = 3)	0.1% (n = 1)	The 3 patients had pancreatic solid lesions as the puncture target: 2 patients experienced acute pancreatitis (moderate: 1, severe: 1) and 1 patient experienced fatal duodenal perforation.
Mekky, M.A.	2010	[1]	141	Gastric submucosal lesions	0.0% (n = 0)	0.0% (n = 0)	No complications.
Hamada, T.	2013	[28]	1135	Submucosal lesions in the esophagus, stomach, duodenum, small intestine, colon, and rectum	0.4% (n = 5)	0.0% (n = 0)	Hemorrhage requiring blood transfusion: 1 case, hemorrhage requiring endoscopic hemostasis: 4 cases. Hemorrhage sites: stomach (n = 4) and duodenum (n = 1).
Iwashita, T.	2015	[29]	100	Mixed solid masses: pancreatic lesions, abdominal or mediastinal lymph nodes, upper intestine lesions, adrenal gland lesions, liver lesions, gall bladder lesions, spleen lesions	0.0% (n = 0)	0.0% (n = 0)	No complications (puncture using 19-gauge needle).
Cheng, B.	2018	[30]	377	Mixed: lesions in the pancreas, abdomen, mediastinum, and pelvic cavity	1.1% (n = 4)	0.0% (n = 0)	Mild hemorrhage: 4 cases (3 from FNA group, 1 from FNB group). Hemorrhage sites in the FNA group: stomach (1 case) and duodenum (2 cases). Hemorrhage site in the FNB group: stomach.
de Moura, D.T.H.	2020	[31]	229	Submucosal lesions	0.4% (n = 1)	0.0% (n = 0)	Hemorrhage requiring endoscopic hemostasis: 1 case in the FNA group.

FNA: fine needle aspiration, FNB: fine needle biopsy.

**Table 2:** Treatments and courses after the diagnosis of needle tract seeding (NTS).

Author [Ref.]	Case				EUS-FNA Methods			
	Age	Sex	Punctured Lesions	Treatment of Punctured Lesions	Puncture Route	Needle Size (Gauge)	No. of Needle Passes	Suction
Hirooka, Y., et al. [59]	57	M	IPMC in the body of the pancreas	Surgery (distal pancreatectomy)	TG	22	3	NA
Shah, J.N., et al. [60]	39	F	Intraperitoneal lymph node metastasis of melanoma	Surgery (lymph node resection)	TG	22	1	10 mL
Paquin, S.C., et al. [61]	65	M	Pancreatic tail cancer	Surgery (distal pancreatectomy)	TG	22	5	5 mL
Doi, S., et al. [62]	70	M	Mediastinal lymph node metastasis of gastric cancer	Chemotherapy + surgery (distal gastrectomy)	TE	19	1	10 mL
Ahmed, K., et al. [63]	79	M	Pancreatic body cancer	Surgery (central pancreatectomy)	TG	NA	NA	NA
Chong, A., et al. [64]	55	F	Pancreatic tail cancer	Surgery (distal pancreatectomy)	TG	22	2	NA
Katanuma, A., et al. [65]	68	F	Pancreatic body cancer	Surgery (distal pancreatectomy)	TG	22	4	NA
Anderson, B., et al. [66]	51	M	Intraperitoneal lymph node metastasis of pancreatic head cancer	Chemoradiotherapy	TG	NA	NA	NA
Ngamruengphong, S., et al. [67]	66	M	Pancreatic body and tail cancer	Surgery (subtotal pancreatectomy)	TG	19, 22	3	NA
	77	F	Pancreatic tail cancer	Surgery (distal pancreatectomy)	TG	19	Cystic region: 1; Solid region: 2	NA
Tomonari, A., et al. [68]	78	M	Pancreatic body cancer	Surgery (distal pancreatectomy)	TG	22	2	NA
Sakurada, A., et al. [69]	87	F	Pancreatic body cancer	Surgery (distal pancreatectomy)	TG	22	3	NA
Minaga, K., et al. [70]	64	F	Pancreatic body cancer	Surgery (distal pancreatectomy)	TG	22	3	10 mL
Minaga, K., et al. [71]	72	F	Pancreatic body cancer	Surgery (distal pancreatectomy)	TG	NA	NA	NA
Kita, E., et al. [72]	68	F	Pancreatic body and tail cancer	Intensity-modulated radiotherapy	TG	22	2	NA
Yamabe, A., et al. [73]	75	M	IPMC in the body of the pancreas	Chemotherapy	TG	25	NA	20 mL
Iida, T., et al. [74]	78	F	Pancreatic body cancer	Surgery (distal pancreatectomy)	TG	22	3	NA
Goel, A., et al. [75]	57	M	Intraperitoneal recurrence of carcinoma of unknown primary	Chemotherapy	TG	19	2	NA
Sakamoto, U., et al. [76]	50	M	Pancreatic tail cancer	Surgery (distal pancreatectomy)	TG	22	2	Slow-pull
Matsumoto, K., et al. [77]	50	M	Pancreatic body cancer	Chemotherapy	TG	21	3	NA
Matsui, T., et al. [78]	68	F	Pancreatic body cancer	Surgery (distal pancreatectomy)	TG	19, 20, 22	4	NA
	70	M	Pancreatic body cancer	Surgery (distal pancreatectomy)	TG	22	1	NA
Yane, K., et al. [79]	47	M	Pancreatic body cancer	Surgery (distal pancreatectomy)	TG	22	4	NA
	78	F	Pancreatic body cancer	Surgery (distal pancreatectomy)	TG	22	4	NA
Rothermel, L.D., et al. [80]	61	M	Pancreatic body cancer	Surgery (distal pancreatectomy)	TG	25	3	NA
Sato, N., et al. [81]	83	F	Pancreatic body cancer	Surgery (distal pancreatectomy)	TG	22	2	NA
Yamaguchi, H., et al. [82]	78	M	SPN in the body of the pancreas	Surgery (distal pancreatectomy)	TG	22	4	0 mL
Okamoto, T., et al. [83]	72	F	Pancreatic tail cancer	Surgery (distal pancreatectomy)	TG	22	5	Slow pull × 3; 10 mL × 1; 5 mL × 1

F, Female; M, Male; NA, Details unknown/not available; TG, Transgastric; TE, Transesophageal.

**Table 3:** Mode of NTS diagnosis.

CT	FDG-PET	UGE	EUS	CA19-9 Increase	UGE Findings	EUS Findings
NE	NE	NE	NE	NE	NE	NE
NE	NE	NE	NE	NE	NE	NE
DA	NE	DA	DA	DA	No ulcers found, and the gastric mucosal surface was normal	We observed a mass inside the stomach wall (diameter 3 cm); the mass extended from the proper muscular layer to the retroperitoneal space
NDA	NE	DA	DA	NE	Well demarcated protrusion (approximately 4 mm in diameter) in the middle part of the esophagus	A hypoechoic region (diameter 8 mm) inside the esophageal wall
DA	DA	DA	DA	NE	Appearance of irregular gastritis, differing from typical gastric cancer	A hypoechoic region, primarily in the proper muscular layer, extending from the mucosal layer to the serous membrane
NDA	DA	DA	DA	DA	Linear mass (4 cm long) on the greater curvature side of the posterior wall of the gastric body	A hypoechoic mass was observed close to the resected pancreatic region, associated with the swelling of surrounding lymph nodes
DA	NE	DA	NE	NE	SMT-like mass in the posterior wall of the upper gastric body	NE
NE	NE	DA	DA	NE	Linear ulcer at the esophagogastric junction	A hypoechoic mass (1 cm in diameter) in the submucosal layer
NE	NE	DA	DA	NE	Wall thickening in the gastric antral region (details unknown)	Wall thickening in the gastric antral region (details unknown)
NE	NE	DA	NE	NE	Recurrent signs in the stomach wall (details unknown)	NE
DA	NE	DA	NE	NE	SMT-like mass in the posterior wall of the gastric body	NE
DA	DA	DA	DA	NDA	SMT-like mass in the posterior wall of the gastric body	A hypoechoic region with cystic lesions
NDA	DA	DA	DA	NE	SMT-like mass (diameter 12 mm) in the posterior wall of the gastric body	An internally heterogeneous hypoechoic mass located primarily in the submucosal layer
NE	NE	DA	NE	NE	3 cm mass with an ulcer in the posterior wall of the gastric body	NE
NE	DA	DA	NE	NE	SMT-like mass in the posterior wall of the gastric body	NE
DA	NE	DA	DA	NE	SMT-like mass in the posterior wall of the gastric body	anechoic region (diameter 24 mm) located primarily in the submucosal layer
NE	DA	DA	DA	NE	SMT-like mass (25 mm in diameter) in the posterior wall of the lower gastric body	Mass extending from the submucosal layer to the proper muscular layer
DA	NE	DA	NE	NE	Ulcerous mass (50 mm in diameter) outside the posterior wall of the cardiac region of the stomach	NE
DA	DA	DA	DA	NE	SMT-like mass, 20 mm in diameter, with irregular mucosa in the posterior wall of the upper gastric body	A hypoechoic mass (diameter 20 mm) located in the submucosal layer
DA	NE	NE	DA	NE	NE	A hypoechoic region extending from the serous membrane to the pancreatic tumor
NE	NE	NE	NE	NE	NE	NE
NE	NE	NE	NE	NE	NE	NE
DA	NE	DA	DA	NE	SMT-like mass in the posterior wall of the gastric body	A hypoechoic mass extending from the submucosal layer to the proper muscular layer
DA	NE	DA	DA	NE	SMT-like mass in the posterior wall of the gastric body	A hypoechoic mass extending from the submucosal layer to the proper muscular layer
NE	DA	DA	NE	DA	Ulcerous protrusion with hemorrhage in the posterior wall of the gastric body	NE
DA	NE	DA	DA	DA	SMT-like mass, 10 mm in diameter, in the posterior wall of the gastric body	A hypoechoic mass in the submucosal layer
DA	DA	DA	NE	NDA	Protruding lesion in the posterior wall of the gastric body	NE
DA	NE	NE	NE	DA	NE	NE

CT; Computed Tomography, EUS; Endoscopic ultrasound, FDG-PET; FluoroDeoxyGlucose-Positron Emission Tomography, NE; Not Entered, UGE; Upper Gastrointestinal Endoscopy, DA; Detection of Abnormality, NDA; No Detection of Abnormality.

**Table 4:** Time, mode, treatment, and post-treatment course after NTS diagnosis.

Time to NTS Diagnosis	NTS Diagnosis Method	Treatment after NTS Diagnosis	Course after NTS Diagnosis
10 days after EUS-FNA (during surgery)	Surgery (during surgery, white nodules 7 mm in diameter on the posterior wall of stomach, and positive for peritoneal lavage cytology)	Distal pancreatectomy + partial gastrectomy	Death 25 months after NTS diagnosis
6 months after EUS-FNA (during surgery)	Surgery (during surgery, a 3 cm black pigmented region was noted on the posterior wall of the stomach)	Lymph node resection + partial gastrectomy	Details unknown
21 months after surgery	EUS-FNA	Chemotherapy	Death 12 months after chemotherapy initiation
18 months after surgery	Endoscopic biopsy	Radiotherapy	Resolution of esophageal lesions 2 months after the initiation of radiotherapy
36 months after surgery	Endoscopic biopsy	Total gastrectomy	Death due to melanoma progression
26 months after surgery	Endoscopic biopsy	Details unknown	Details unknown
22 months after surgery	Endoscopic biopsy	Chemotherapy	Death 10.8 months after NTS diagnosis
Details unknown	EUS-FNA	Details unknown	Details unknown
27 months after surgery	Endoscopic biopsy	Details unknown	Details unknown
26 months after surgery	Endoscopic biopsy	Details unknown	Details unknown
28 months after surgery	Endoscopic biopsy	Subtotal gastrectomy	Death 24.9 months after NTS diagnosis
19 months after surgery	EUS-FNA	Partial gastrectomy	Survival 62.4 months after NTS diagnosis
8 months after surgery	EUS-FNA	Partial gastrectomy	Details unknown
24 months after surgery	Endoscopic biopsy	Gastrectomy	Details unknown
7 months after EUS-FNA	Endoscopic biopsy	Details unknown	Details unknown
3 months after EUS-FNA	EUS-FNA	Chemotherapy	Death 26 months after NTS diagnosis
6 months after surgery	Details unknown	Distal gastrectomy	Survival 40.5 months after NTS diagnosis
11 months after EUS-FNA	Endoscopic biopsy	Details unknown	Details unknown
24 months after surgery	Endoscopic biopsy	Partial gastrectomy	Details unknown
8 months after EUS-FNA	Surgery	Distal pancreatectomy + partial gastrectomy	Details unknown
25 days after EUS-FNA (during surgery)	Surgery (during surgery, hard tumor was found in the posterior wall of the gastric body)	Distal pancreatectomy + partial gastrectomy	Recurrence leading to death 18 months after NTS diagnosis
113 days after EUS-FNA (during surgery)	Surgery (during surgery, hard tumor was found in the posterior wall of the gastric body)	Distal pancreatectomy + partial gastrectomy + resection of mesenterium of the small intestine	Survival 18 months after NTS diagnosis
27.8 months after surgery	Details unknown	Surgery (details unknown)	Death 17.4 months after NTS diagnosis
34.9 months after surgery	Details unknown	Surgery (details unknown)	Survival 4.6 months after NTS diagnosis
42 months after surgery	Endoscopic biopsy	Chemotherapy + radiotherapy + gastrectomy	Survival 72 months after initial DP
25 months after surgery	EUS-FNA	Partial gastrectomy + lymph node resection	Recurrence-free survival 5 months after NTS diagnosis
67 months after surgery	Surgery	Distal gastrectomy	Details unknown
4 months after EUS-FNA (during surgery)	Surgery (positive for peritoneal lavage cytology)	Distal pancreatectomy + partial gastrectomy + chemotherapy	Death 5 months after NTS diagnosis

EUS-FNA; Endoscopic Ultrasound-Guided Fine-Needle Aspiration, NTS; Needle Tract Seeding.
